# Mortality of chronic disorders of consciousness in adults and adolescents – a retrospective community based study from Salzburg, Austria

**DOI:** 10.3389/fneur.2024.1465564

**Published:** 2024-12-02

**Authors:** Laura Schnetzer, Tanja Prüwasser, Jürgen Bergmann, Georg Zimmermann, Martin Kronbichler, Stefan Leis, Eugen Trinka

**Affiliations:** ^1^Department of Neurology, Neurological Intensive Care and Neurorehabilitation, Christian Doppler Medical Centre, Centre for Cognitive Neuroscience Salzburg, Member of the European Reference Network EpiCARE, Paracelsus Medical University, Salzburg, Austria; ^2^Neuroscience Institute, Christian Doppler Medical Centre, Centre for Cognitive Neuroscience Salzburg, Paracelsus Medical University, Salzburg, Austria; ^3^Karl Landsteiner Institute of Neurorehabilitation and Space Neurology, Salzburg, Austria; ^4^Spinal Cord Injury and Tissue Regeneration Centre, Paracelsus Medical University, Salzburg, Austria; ^5^Vorarlberg Institute for Vascular Investigation and Treatment (VIVIT), Feldkirch, Austria; ^6^Department of Mathematics, Paris Lodron University, Salzburg, Austria; ^7^IDA Lab Team Biostatistics and Big Medical Data, Paracelsus Medical University, Salzburg, Austria; ^8^Department of Artificial Intelligence and Human Interfaces, Faculty of Digital and Analytical Sciences, Paris Lodron University, Salzburg, Austria; ^9^Research Programme Biomedical Data Science, Paracelsus Medical University, Salzburg, Austria; ^10^Department of Psychology, Centre for Cognitive Neuroscience, University of Salzburg, Salzburg, Austria

**Keywords:** minimally conscious state, unresponsive wakefulness syndrome, incidence, survival, epidemiology, Kaplan–Meier curve, etiology, death

## Abstract

**Introduction:**

Epidemiological data on disorders of consciousness (DoC) is rare and very heterogeneous due to difficulties in case ascertainment and differences in health care pathways between countries. This study reports data on mortality and survival time for DoC patients in Salzburg, Austria.

**Methods:**

All patients with DoC were registered in the health care region of Salzburg North, Austria between 2007 and 2022 and their death data retrieved from the Statistik Austria. The 1- and 5-year mortality was calculated, also in relation to several explanatory variables (age, sex, etiology, diagnosis, CRS-R score, improvement). Furthermore, the incidence, survival functions using the Kaplan–Meier estimator and a Cox-Regression were calculated.

**Results:**

The mean annual incidence is 2.2 DoC/100.000 inhabitants in the Salzburg North region. The crude 1- and 5-year mortality rates were 25.9 and 55.1%, respectively, and the median survival of DoC patients based on the Kaplan–Meier estimator was 6.3 years. Moreover, the mortality was lower in women and in younger patients, those of traumatic etiology, and those with higher CRS-R scores, better diagnosis or an improvement of diagnosis until discharge from hospital.

**Conclusion:**

This article gives a rare insight into epidemiological data on DoC and shows which factors influence the mortality of these patients. Moreover, it is the first community based study on mortality of DoC in Salzburg, Austria.

## 1 Introduction

Disorders of Consciousness (DoC) are one of the most severe chronic neurological disorders, resulting from a traumatic or non-traumatic brain injury. The unresponsive wakefulness syndrome (UWS (1), formerly known as vegetative state (2) or apallic syndrome (3)) is the most severe form, where the patient opens the eyes but shows no awareness of him- or herself or the surroundings. The minimally conscious state (MCS (4)) is the first remission stage when the patient shows the first signs of consciousness like eye tracking. The most commonly used scale for grading DoCs is the Coma Recovery Scale-Revised (CRS-R (5)), a standardized bedside evaluation tool, but further diagnostics such as fMRI (6) and EEG have gained importance in recent years to correctly diagnose patients suffering from cognitive motor dissociation (CMD (7)) and are not able to reveal their consciousness at bedside examinations.

Data on the prevalence, incidence and mortality of DoC is rare. Differing nomenclature and timeline definitions, changes in medical practice, the difficulty of case ascertainment in different care situations and differences between countries, complicate the estimates of incidence and prevalence (8, 9). A Polish study found that 7.4% of all patients who were discharged from an ICU had MCS or UWS (10). It was estimated in 2005 that the incidence of vegetative state lies between 0.5 and 2.5 patients/100.000 inhabitants with higher numbers in the US than the UK (8). In Austria, Stepan et al. conducted a point prevalence study of the apallic syndrome in the Vienna region in 2001 with the result of 1.9 apallic patients/100000 inhabitants (11). A follow up study conducted in 2003, resulted in an insignificantly different prevalence of 1.7/100000 inhabitants (12). Another Austrian study conducted between 2007 and 2009 showed a prevalence of 3.36 UWS and 1.5 MCS patients/100000 inhabitants in long-term care facilities (13). A French study from 2010 reported a permanent *VS*/MCS prevalence of 2.8/100000 inhabitants (14). Dutch studies however, only showed a prevalence of 0.1–0.2/100000 inhabitants in the Netherlands (15, 16). Beljaars et al. conclude, that the different prevalences reported for the two regions are a result of differing social values of the populations. While in the Netherlands life prolonging measures are seen as “futile” at some point, patients in Vienna (and probably throughout Austria) are “seen as severely disabled” and the “preservation of human life is seen as essential” (17). Two systematic reviews on prevalence studies in DoC found a high heterogeneity which rendered summarizing or comparing these studies impossible and therefore, articulated the need for further studies (18, 19). It was also stated that only a very limited range of countries conducted prevalence studies (19).

Whereas mortality is high in the first year after the onset of DoC, it decreases thereafter (9). Strauss found in 1999 that the mortality rate fell by 8% for each year after the onset. For a 15 year old patient, 1 year after onset, they calculated a life expectancy of 10.5 years (20). A 5 year follow-up study of patients in persistent vegetative state conducted between 1973 and 1978, recorded the death of 75% of all patients within this timeframe (21). In 2022 a mortality rate of 28.7% after 2 years was described for DoC patients (22). Survival rates of 80.5, 72 and 69.7% were reported for 1, 3 and 8 years after onset in 2023 (23). A recent systematic review found a pooled mortality rate of DoC patient of anoxic etiology of 26% in a median time of 16 months after onset (24).

This study aims to report on incidence and mortality of DoC patients in Salzburg, Austria.

## 2 Methods

All patients who suffered from a disorder of consciousness and were treated at the Department of Neurology, Neurological Intensive Care and Neurorehabilitation at the Christian Doppler Medical Center in Salzburg between the years 2007 and 2022 were identified using an electronic record system. All patients with a diagnosis of UWS or MCS according to the CRS-R were included. The institution is the only Neurology Service in the Region Salzburg Nord (Versorgungsregion VR51) and all patients with DOC at the age of 15 or older in the region are seen there. DoC patients below the age of 15 are not treated at the Department of Neurology and therefore not included. Patients who were admitted for a re-evaluation of their consciousness status and had no data of the time < 6 months after onset available (i.e., received initial treatment after the acute event at another clinic), were excluded as they already survived the critical time period of the first months after onset and would distort the mortality and survival time results. Patients who died or recovered full consciousness within the acute timeframe (<28 days), were also excluded as this study focuses on the more homogenous population of prolonged and chronic DoC patients to make results comparable. Furthermore, those patients whose onset of DoC occurred after the censoring date, which was set at the 31.12.2022 were excluded. This was the time point until which the latest death statistics were available. Finally, patients who are resident outside of Austria, who are not of Austrian nationality, had to be excluded as they are not captured by the Austrian death statistics (lost to follow-up).

Birth date, date of onset of DoC and etiology were documented and mortality data were retrieved from the “Statistik Austria” (Bundesanstalt Statistik Österreich, Guglgasse 13, 1110 Wien, Austria), a governmental institution, after receiving the ethics approval (No. 1148/2021). The mortality data included all deaths that occurred until the 31.12.2022 (censoring date). The first CRS-R score and diagnosis after onset and the last before discharge were documented.

For the data collection, Excel (25) was used. For the statistical analysis and creation of figures, R (26) was used, with the most important packages being ggplot2 (27), survival (28) and survminer (29). A level of significance of *p* ≤ 0.05 was applied. The incidence was calculated for the care region Salzburg North (VR 51 including the districts Salzburg city, Salzburg surrounding area and Hallein). The cases from Salzburg South (VR 52 including the regions Tamsweg, St Johann im Pongau and Zell am See) and other regions were not considered for population based incidence rates due to selection bias. As no patients below the age of 15 are included in this study, this age group was also not included in the incidence calculations. The mortality is described as 1- and 5- year mortality, also in relation to several prognostic variables age, age groups at onset (15–29, 30–44, 45–59, ≥60), sex, etiology (traumatic, hypoxic and other non-traumatic etiologies), first diagnosis and CRS-R score after onset and last ones before discharge (diagnosis was defined according to the CRS-R score as UWS, MCS or recovered), epilepsy/status epilepticus (according to medical records) as well as improvement or deterioration of diagnosis and CRS-R score (an improvement of diagnosis was defined as the change of diagnosis from UWS to MCS, or recovered, or from MCS to recovered; the deterioration as a change from recovered to MCS or UWS, or from MCS to UWS; an improvement of the CRS-R score defined as an increase of the total score, the deterioration as the decrease of the total CRS-R score). To measure the association between the variables and the outcome, the Odds Ratio (OR) was used. For the standardization of the mortality rate, a reference population was chosen. The median of DoC onset in the patient group was the year 2013, and as the study was conducted in Salzburg, the population of the Country of Salzburg in 2013 was used as reference population. The number of deaths in 2013 were used as reference for the 1-year mortality calculations and the deaths from 2013 to 2017 were added for the 5-year mortality calculations. The age classes of 15–29, 30–44, 45–59, ≥60 were included (no DoC patient was below the age of 15, therefore the age group <15 was dismissed in the reference population as well). The population numbers of Salzburg at the beginning of 2013 and the number of deaths from 2013 to 2017 were extracted from the open access database of the Statistik Austria. For the calculation of the standardized mortality ratio (SMR), the Vandenbroucke method (30) was applied.

Furthermore, survival functions were estimated using Kaplan–Meier estimator to illustrate the survival times, and based on these estimates, the median survival was calculated. All assumptions underlying the Kaplan–Meier estimator were met. 72 patients had to be censored for these calculations as they were still alive at the censoring date. Not only the DoC patient group as a whole was analyzed, but the survival functions were also estimated for different subgroups (according to sex, age group at onset, etiology, first diagnosis and CRS-R score after onset and last one before discharge, improvement or deterioration of the diagnosis until discharge).

Finally, a Cox-Regression, including the estimation of hazard ratios (HR) was performed, in order to further evaluate the influence of certain variables on the survival time simultaneously. The following predictors were used: age at onset, sex, etiology, first CRS-R score and difference of first and last CRS-R score. Using a Schoenfeld test, the compliance with the requirements of the proportional hazards model was ensured and *p*-values were adjusted according to Bonferroni-Holm.

## 3 Results

**Figure 1:**
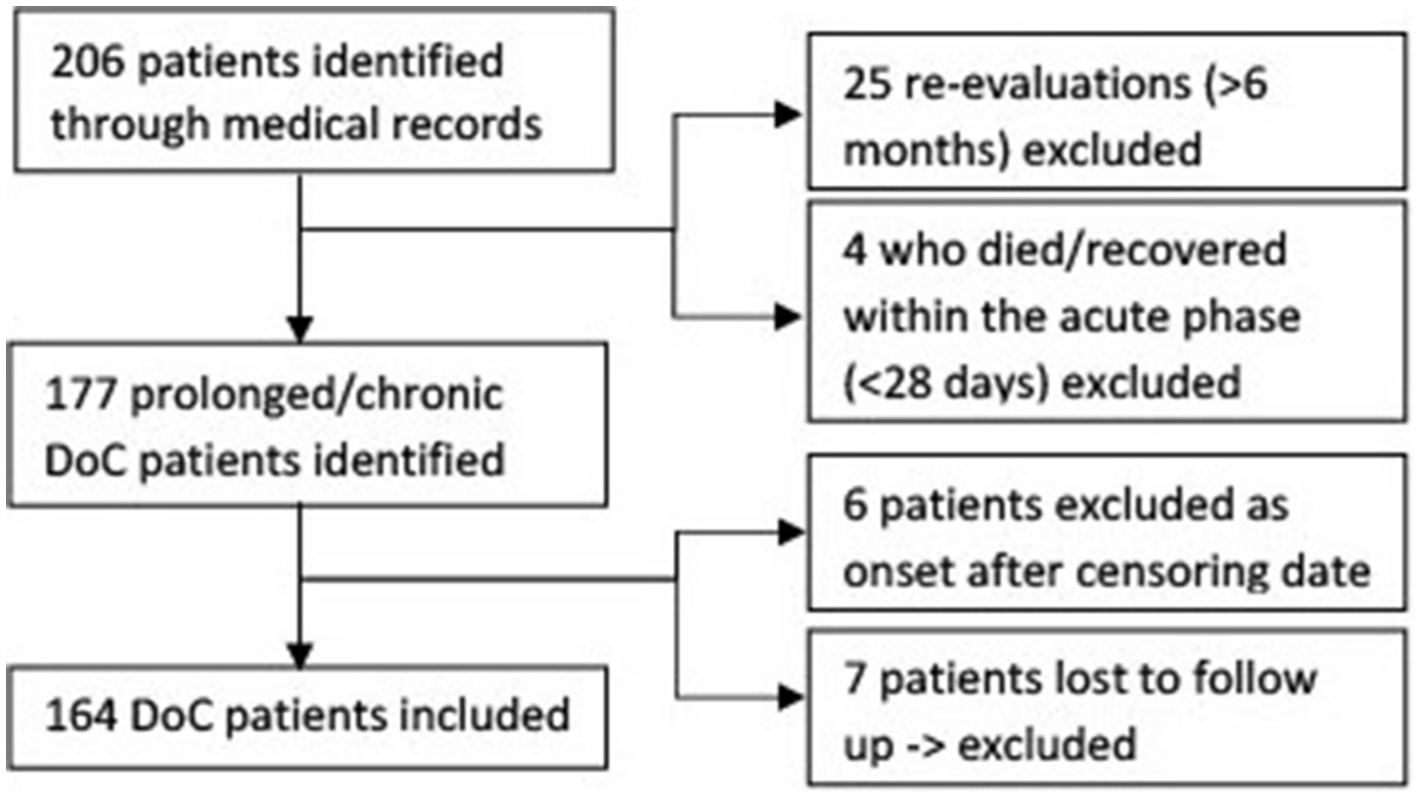
Flow chart of in- and excluded disorders of consciousness patients.

206 DoC patients could be identified. 164 patients could be included in the study, the exact exclusion process can be seen in Figure 1. The values of the reference population for the standardization of the mortality rate can be found in Table 1.

**Table 1:** Reference population: population and deaths in Salzburg County 2013–2017.

Age group	Deaths (2013, women)	Deaths (2013–2017, women)	Women total	Deaths (2013, men)	Deaths (2013–2017, men)	Men total
15–29	9	35	48,959	26	131	50,455
30–44	32	156	55,957	45	249	54,907
45–59	126	608	61,334	221	1,148	58,319
≥60	2073	10,672	68,613	1862	9,301	53,793

Source: extracted from Statistik Austria (43).

Of the 164 patients included, 51 (31%) were women and the median age at DoC onset was 53 years (range 17–91 years). 75% of all patients were above the age of 45 years. 48 (29%) patients had a hypoxic, 57 (35%) a traumatic and 59 (36%) another etiology (ischemic strokes, encephalitis or hemorrhages), details can be found in Table 2.

**Table 2:** DoC etiologies.

Etiology	Number of patients (%)
Traumatic	57 (34.8%)
Hypoxic	48 (29.3%)
Hemorrhages	23 (14%)
Ischemic stroke	18 (11%)
Encephalitis	7 (4.3%)
Metabolic encephalopathy	5 (3.1%)
Mixed encephalopathy, malign neuroleptic syndrome, brain tumor, intoxication, toxic shock syndrome, normal pressure hydrocephalus	1 (0.6%) each

The median time from DoC onset to the first CRS-R evaluation and diagnosis was 52 days (range 8–183 days, IQR 33–75), the median time until discharge was 5 months (range 0–42 months). During the hospital stay, 65 patients (41%) improved their category of DoC, 3 (2%) deteriorated and 89 (57%) showed neither (for 7 patients first and/or final diagnosis was missing). At the time of discharge 59 patients (36%) were diagnosed with UWS, 46 (28%) with MCS and 57 (35%) had recovered their consciousness (for 2 patients no diagnosis at discharge was available). While the median of the first CRS-R scores after onset was 5 (range 0–23), the median CRS-R score at discharge was 11 (range 1–23). 69 patients (42%) had structural (symptomatic) epilepsy or a status epilepticus during the hospital stay.

92 of the 164 DoC patients (56%) died during the study period (until 31.12.2022). For 24 patients external (injuries) and natural causes of death were described, for the other 68 only natural causes of death were reported. The exact ICD-10 codes of the causes of death provided by the Statistik Austria are listed in Table 3. For those who died, the median time from DoC onset until death was 391 days (range 39–4,344 days). 45.2% of the deceased patients died within the first year after onset of DoC. At time of death, the median age was 60 years (range 19–91 years).

**Table 3:** ICD-10 codes of causes of death of disorders of consciousness patients who deceased during the study period (*n* = 92, for 24 cases 2 ICD-10 codes were reported) including the number of cases in brackets if more than one case occurred.

ICD-10 codes (*n*)		
B Infectious disease		24
C D	Neoplasms	349, 539, C55, 921, 961
		432
E Metabolic disease		109, 119, 669
F Substance use		102 (2), 192
G Disease of the nervous system		08, 009, 10, 309, 903, 931, 938 (4), 959
I Disease of the circulatory system		10, 251, 255, 258 (3), 269 (2), 420 (2), 469 (2), 490 (2), 509 (3), 519, 609 (5), 615, 619, 620, 639, 64 (2), 691, 694
J Disease of the respiratory system		189, 411 (4), 449, 690
K Disease of the digestive system		228, 432, 729, 801
N Disease of the genitourinary system		19, 390
Q Congenital malformation		211
S T W X Y	Injuries, external causes	062, 069 (11), 399
		814, 861, 905 (2), 913, 981 (4), 983
		108, 109
		591, 599 (10)
		830, 848, 850, 859, 870 (2), 883, 899 (3)
U SARS-Cov-2		50

The mean annual incidence rate of DoC in the Salzburg North region between the years 2007 and 2022 was 2.2 with a 95% confidence interval (CI) of [1.6–2.8] /100.000 inhabitants at the age of 15 or older. Considering the diagnosis at discharge from the hospital, in the Salzburg North region the mean annual incidence rate was 0.6 MCS, 95% CI [0.3–0.9] and 0.8 UWS patients, 95% CI [0.5–1.1] /100.000 inhabitants aged 15 years or older. Selection bias has to be taken into account for the Salzburg South region, as it is possible that not all DoC patients have been transferred to the Christian Doppler Medical Center and therefore were not considered for population based incidence rates.

Two patients had to be excluded concerning the 1-year mortality calculations due to censoring, therefore 162 patients could be included. For the 5-year mortality calculations, 26 patients were censored and 138 included. 72 (44%) patients survived until the end of the study period, so their survival time had to be censored with the 31.12.2022.

**Figure 2:**
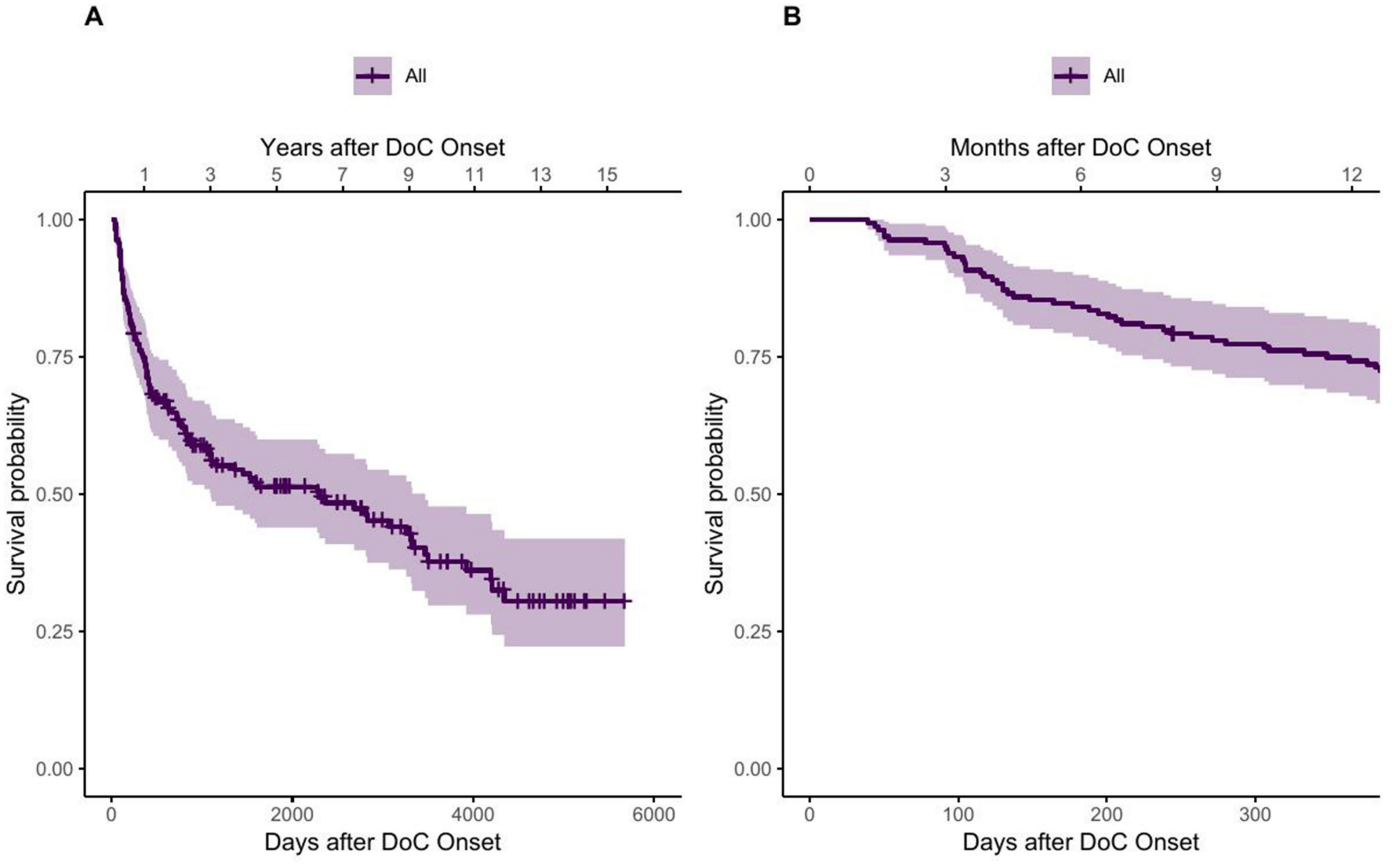
Survival function of DoC patients using the Kaplan–Meier estimator. **(A)** showing the whole observation period and **(B)** showing the first year after onset.

42 patients died within 1 year after onset, whereas 120 patients survived the first year. The 1-year crude mortality rate therefore was 26%. 76 patients had died and 62 patients were still alive 5 years after DoC onset, which corresponds to a crude mortality rate of 55%. When calculating the mortality corresponding to the reference population we obtain a 1-year mortality of 9 and a 5-year mortality of 17 per 100,000 persons for the whole study duration of 16 years. Complementing the crude mortality rates, Figure 2 shows the estimated survival function of all DoC patients using the Kaplan–Meier estimator, thereby also taking censoring into account. The median survival was 2,299 days, 95% CI [1097–3,468], which equals 76 months, 95% CI [36–114] or about 6.3 years, 95% CI [3–9.5] after DoC onset.

The steep first part of the survival function of Figure 2 suggests that the probability of dying increases rapidly in the first time after onset of DoC. This observation is confirmed by the quantiles of the survival function, listed in Table 4, as fewer days lie between the first quantiles than between the following ones.

**Table 4:** Quantiles for survival probability in days and years for DoC patients (NA, not available).

Survival probability	Quantile in days	Quantile in years
0.95	92 (50, 115)	0.25 (0.14, 0.32)
0.9	117 (97, 177)	0.32 (0.27, 0.49)
0.85	164 (123, 271)	0.45 (0.34, 0.74)
0.8	238 (164, 384)	0.65 (0.45, 1.05)
0.75	348 (224, 522)	0.95 (0.61, 1.43)
0.7	413 (309, 818)	1.13 (0.85, 2.24)
0.6667	628 (381, 1,028)	1.72 (1.04, 2.82)
0.65	671 (391, 1,106)	1.84 (1.07, 3.03)
0.6	840 (628, 2,276)	2.3 (1.72, 6.23)
0.55	1,361 (818, 2,830)	3.73 (2.24, 7.75)
0.5	2,299 (1,097, 3,468)	6.29 (3, 9.5)
0.45	3,068 (1,586, 4,209)	8.4 (4.34, 11.52)
0.4	3,468 (2,683, NA)	9.5 (7.35, NA)
0.35	4,195 (3,314, NA)	11.49 (9.07, NA)
0.3333	4,209 (3,325, NA)	11.52 (9.1, NA)
0.3	NA (3,501, NA)	NA (9.59, NA)
0.25	NA (4,209, NA)	NA (11.52, NA)

### 3.1 Age

**Figure 3:**
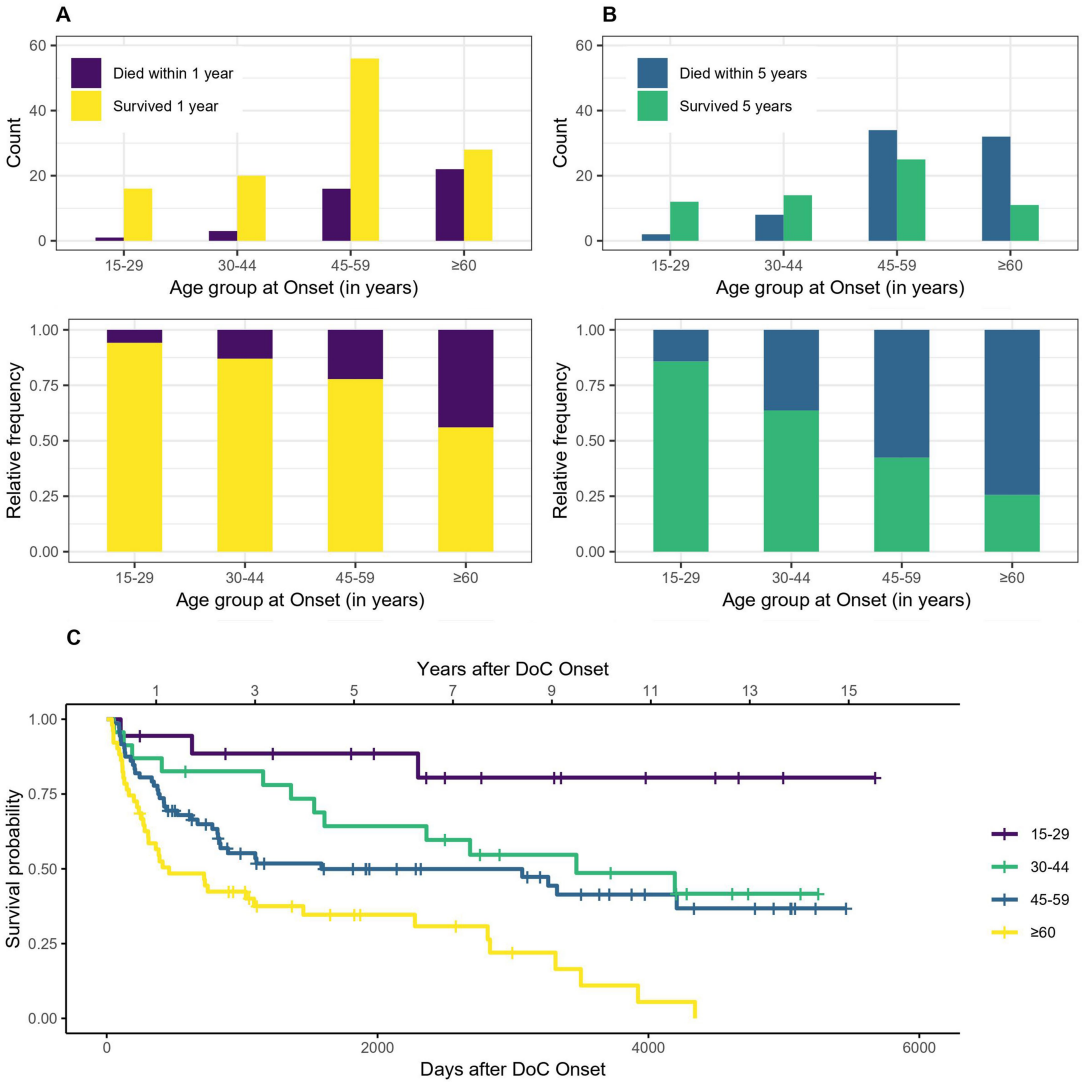
Survival according to age group: absolute and relative count of persons surviving 1 **(A)** or 5 **(B)** years after onset of DoC, **(C)** Kaplan–Meier Survival Function according to age group (no confidence intervals are shown due to the high number of groups).

Regarding the survival according to different age groups (15–29, 30–44, 45–59, ≥60 years), a clear trend of better survival of persons that are younger at DoC onset can be observed (see Figure 3). The median age at DoC onset of patients who survived the first year is 51 years (range 17–77 years), whereas it is 60.5 years (range 29–91 years) for those who died within the first year after DoC onset. For the 5 years estimate it is 47 (range 18–77 years) and 57 years (range 17–91 years) respectively. 94% of patients aged 15–29 years survived the first year after DoC onset, whereas only 56% of those aged >60 years did. For the 5 years estimate it was 86 and 26%, respectively. The median survival is 3,468 days, 95% CI [1,608, not possible to estimate - NA] (114 months, 95% CI [53-NA] or 9.5 years, 95% CI [4.4-NA]) after DOC onset for patients aged 30–44, 1,586 days, 95% CI [822-NA] (52 months, 95% CI [27-NA] or 4.4 years, 95% CI [2.3-NA]) for patients aged 45–59 and 461 days, 95% CI [306–2,276] (15 months, 95% CI [10–75] or 1.26 years, 95% CI [0.8–6.2]) for patients aged ≥60. No median survival can be estimated for the patients aged 15–29, as the Kaplan–Meier estimator does not fall below 50%.

### 3.2 Sex

**Figure 4:**
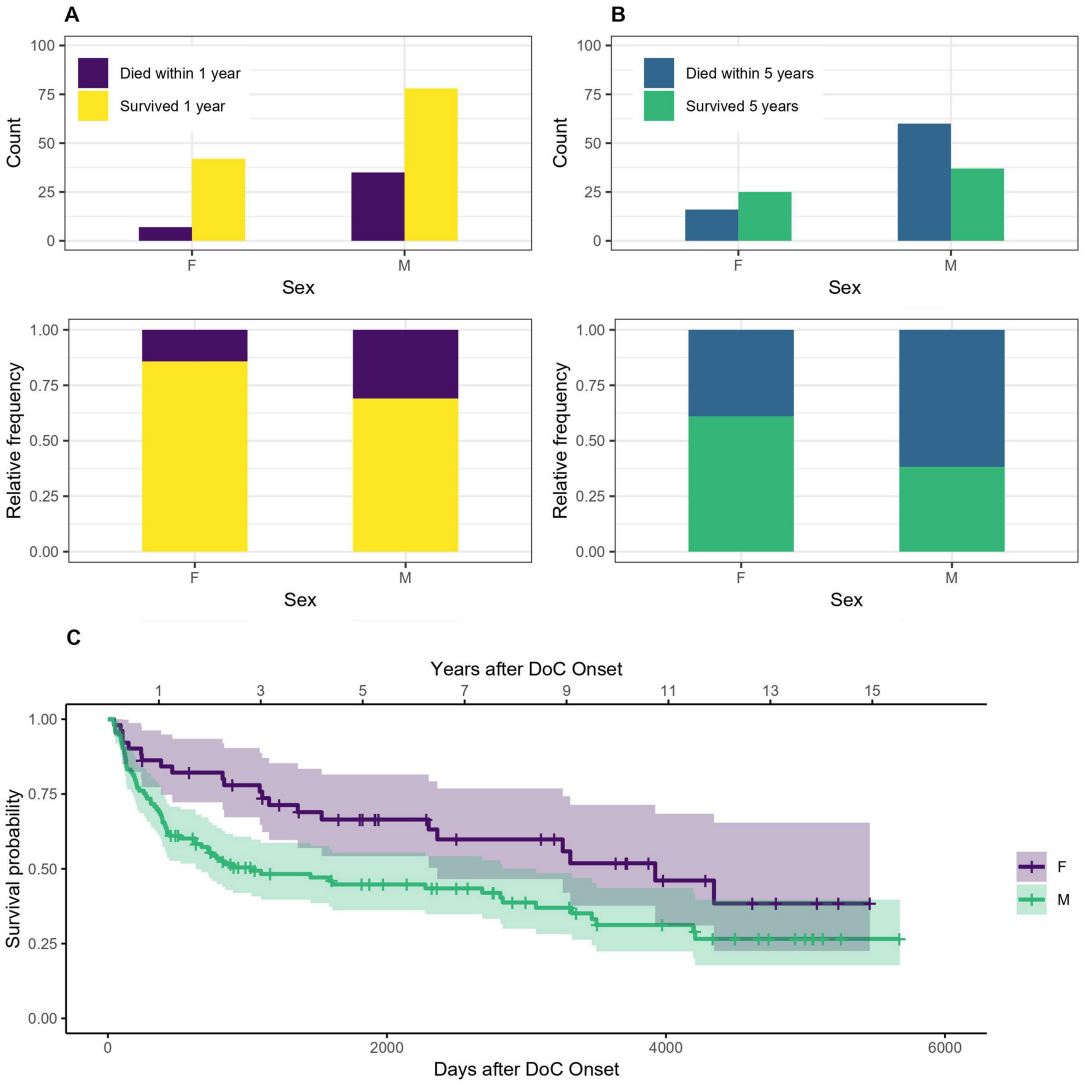
Survival according to sex: absolute and relative count of women and men surviving 1 **(A)** or 5 **(B)** years after onset of DoC, **(C)** Kaplan–Meier Survival Function according to sex (W, women; M, men).

Figure 4 indicates that women tend to have a lower probability of dying after 1 or 5 years in DoC than men. The OR for dying within the first year is 0.37, 95% CI [0.15–0.91] for woman compared to men, which means that women have a 63% lower probability of dying within the first year after DoC onset compared to men. The OR for dying within 5 years after DoC onset is 0.40, 95% CI [0.19–0.84] for women compared to men, so their probability of dying within the first 5 years after DoC onset is 60% lower. The median survival amounts to 3,923 days, 95% CI [2361-NA] (129 months, 95% CI [78-NA] or 10.8 years, 95% CI [6.5-NA]) after DoC onset for women and 1,028 days, 95% CI [630–3,068] (34 months, 95% CI [21–101] or 2.8 years, 95% CI [1.7–8.4]) after DoC onset for men.

An indirect approach is used to establish the SMR according to age and sex. The expected 1-year mortality rate is 0.011933 (*100000), therefore the standardized mortality quotient is 21.73, 95% CI [15.65–28.79]. While 42 deaths occurred in the patient group within 1 year after onset, 1.93 would have been expected according to the reference population. Using the 5-year mortality data, we receive an expected mortality rate of 0.0604592 (*100000) and a standardized mortality quotient of 9.11, 95% CI [7.18–11.72]. While 76 deaths occurred in the patient group within 5 years after onset, 8.34 would have been expected according to the reference population.

### 3.3 Etiology

**Figure 5:**
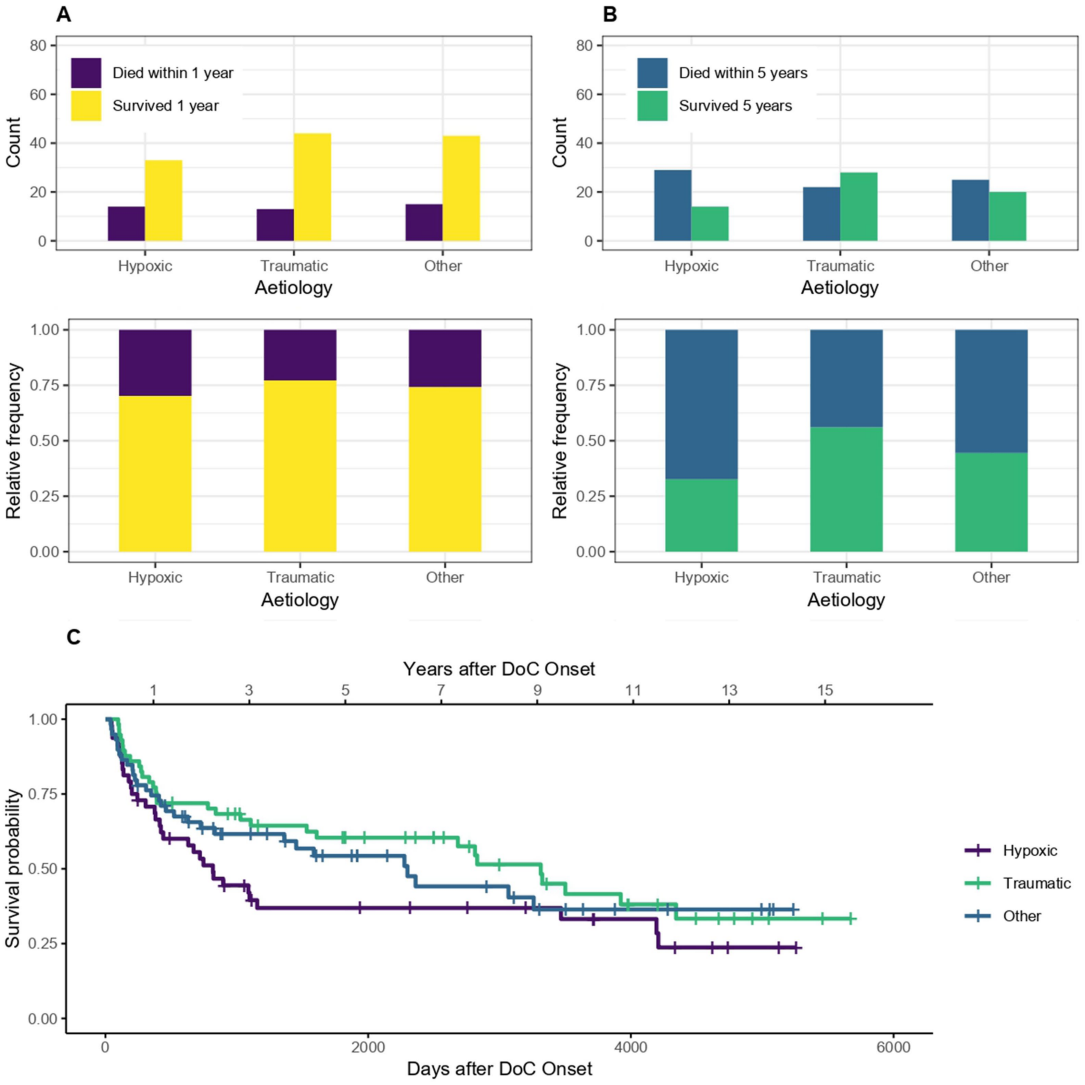
Survival according to etiology: absolute and relative count of DoC patients of traumatic and non-traumatic etiology surviving after 1 **(A)** or 5 **(B)** years after onset of DoC, **(C)** Kaplan–Meier Survival Function according to etiology.

The survival according to etiology (traumatic, hypoxic and other non-traumatic) is shown in Figure 5. Patients with DoC of traumatic etiology tend to have a lower probability of dying within 5 (but not one) years after DoC onset compared to patients whose DoC was caused by a hypoxic or other non-traumatic brain injury. The sample OR for dying within the first year after DoC onset was 0.67, 95% CI [0.31–1.43] for traumatic compared to non-traumatic cases. 1 year survival was 77% in patients with traumatic DoC and 70% with hypoxic and 74% with other non-traumatic etiologies. The 5 year survival was 56, 33 and 44%, respectively. For 5 years the OR was 0.44, 95% CI [0.21–0.88] and therefore the chances of dying within 5 years after DoC onset was 56% lower for patients of traumatic versus non-traumatic etiology. The median survival was 3,314 days, 95% CI [1608-NA] (109 months, 95% CI [53-NA] or 9.1 years, 95% CI [4.4-NA]) after DoC onset for patients with traumatic etiology, 818 days, 95% CI [422–4,195] (27 months, 95% CI [14–138] or 2.2 years, 95% CI [1.2–11.5]) for patients with hypoxic etiology and 2,299 days, 95% CI [1,361, NA] (76 months, 95% CI [45-NA] or 6.3 years, 95% CI [3.7-NA]) for those with other non-traumatic etiologies.

### 3.4 First diagnosis and CRS-R score

**Figure 6:**
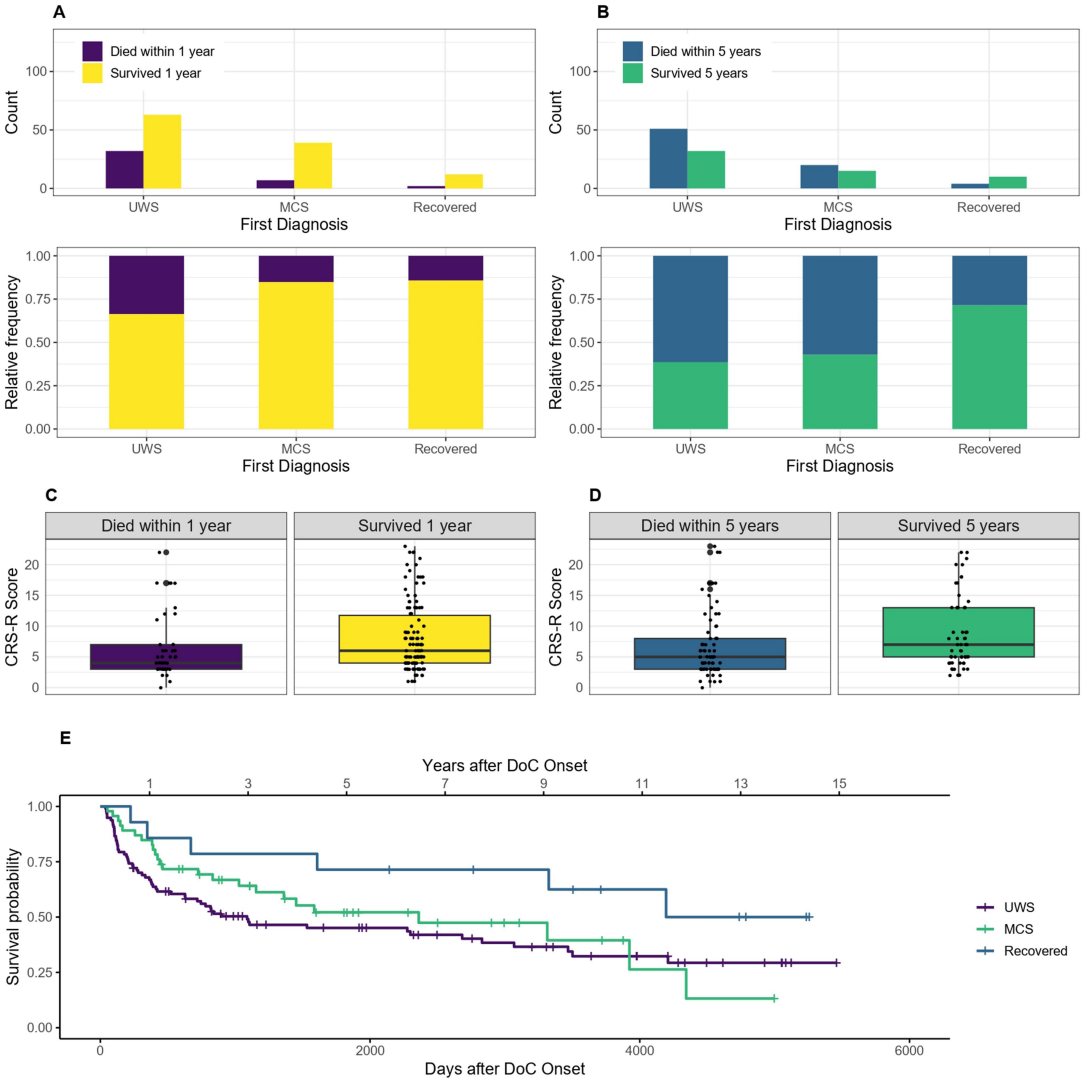
Survival according to first diagnosis and CRS-R score: absolute and relative count of patients with a first diagnosis of UWS, MCS and recovered surviving after 1 **(A)** or 5 **(C)** years after onset of DoC, the survival according to CRS-R score is also shown for 1 **(B)** and 5 **(D)** years, **(E)** Kaplan–Meier Survival Function according to first diagnosis.

Of the 162 patients included for the 1-year mortality calculations, seven had no initial CRS-R score and diagnosis (UWS, MCS or recovered, 1 of them died within 1 year) and therefore had to be excluded from these calculations. Of the 138 patients included for the 5-year mortality calculations, six had no CRS-R score and diagnosis (1 of them died) and were excluded. For the Kaplan–Meier estimator, the seven patients without CRS-R score and diagnosis had to be excluded as well. The median survival time was 1,088 days, 95% CI [630–3,068] (36 months, 95% CI [21–101] or 3 years, 95% CI [1.7–8.4]) after DoC onset for patients with the initial diagnosis of UWS and 2,361 days, 95% CI [1155-NA] (78 months, 95% CI [38-NA] or 6.5 years, 95% CI [3.2-NA]) for patients initially in MCS. As the survival probability for the initial diagnosis recovered did not drop below 50% in this sample, no median survival can be estimated for this group. After 1 year, 86% of recovered, 85% of MCS and 66% of UWS patients (initial diagnosis) were still alive. After 5 years it was 71, 43 and 39%, respectively. Figure 6 shows that patients who are initially diagnosed as UWS tend to have a lower 1- and 5-year survival probability than MCS and recovered patients.

### 3.5 Diagnosis and CRS-R score at discharge from hospital

**Figure 7:**
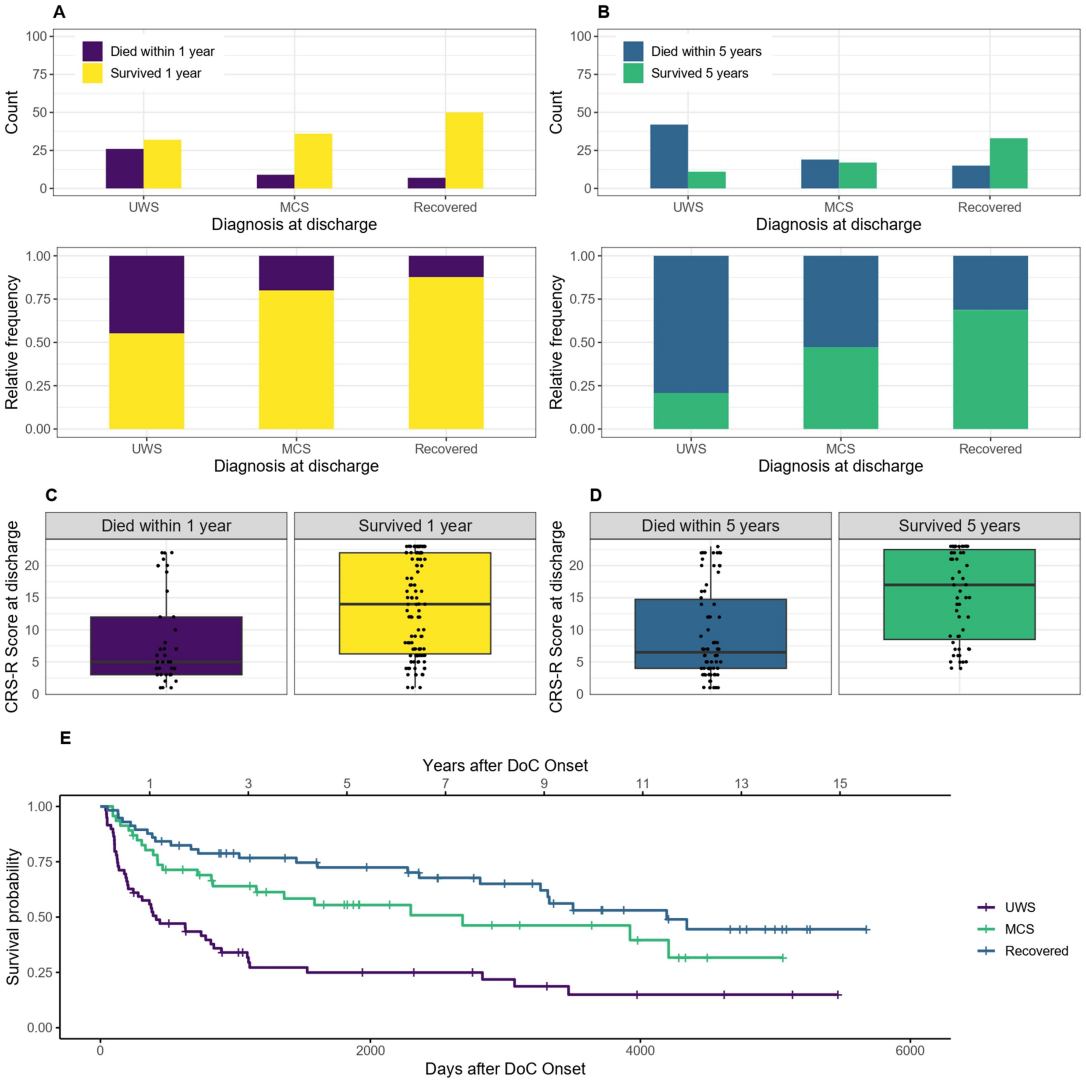
Survival according to diagnosis and CRS-R score at discharge: absolute and relative count of patients diagnosed as UWS, MCS and recovered at discharge surviving after 1 **(A)** or 5 **(C)** years after onset of DoC, the survival according to CRS-R score is also shown for 1 **(B)** and 5 **(D)** years, **(E)** Kaplan–Meier Survival Function according to diagnosis at discharge.

Of the 162 patients included for the 1-year mortality calculations, 2 had no diagnosis and 14 no CRS-R score at discharge (0 and 4 of them died) and therefore had to be excluded from these calculations. Of the 138 patients included for the 5-year mortality calculations, 1 had no diagnosis and 13 no CRS-R score (0 and 6 of them died) and were excluded. For the Kaplan–Meier estimator, the 2 patients without diagnosis at discharge had to be excluded as well. The median survival of patients with the diagnosis UWS at discharge was 413 days, 95% CI [243–896] (14 months, 95% CI [8–29] or 1.1 years, 95% CI [0.7–2.5]) after DoC onset, with MCS 2683 days, 95% CI [1155-NA] (88 months, 95% CI [38-NA] or 7.4 years, 95% CI [3.2-NA]) and for patients who recovered until discharge 4,195 days, 95% CI [3260-NA] (138 months, 95% CI [107-NA] or 11.5 years, 95% CI [8.9-NA]). After 1 year 88% of recovered, 80% of MCS and 55% of UWS patients (diagnosis at discharge) were still alive. After 5 years it was 69, 47 and 21%, respectively. Figure 7 shows the trend that the worse the diagnosis at discharge, the lower the 1- or 5- year survival probability of DoC patients.

### 3.6 Improvement of DoC category and change of CRS-R score

**Figure 8:**
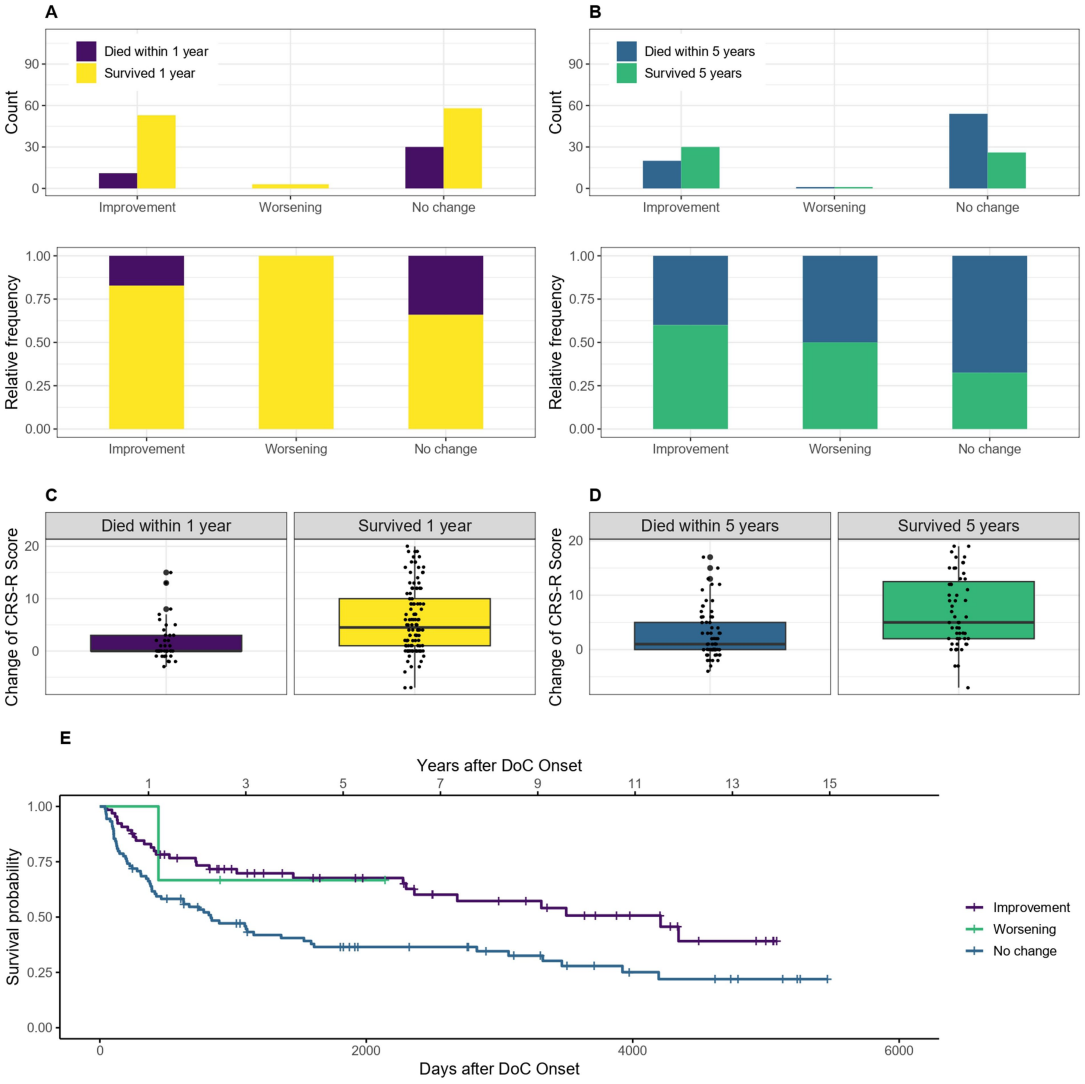
Survival according to improvement/deterioration during hospital stay: absolute and relative count of patients who improved/deteriorated or did not change their diagnosis during the hospital stay surviving after 1 **(A)** or 5 **(C)** years after onset of DoC, the change of CRS-R score points is also shown according to survival of 1 **(B)** and 5 **(D)** years, **(E)** Kaplan–Meier Survival Function according to improvement/deterioration during hospital stay.

Of the 162 patients included for the 1-year mortality calculations, seven had first, last or both diagnoses and 14 one or both CRS-R scores missing (1 and 4 of them died within the first year) and therefore had to be excluded from these calculations. Of the 138 patients included for the 5-year mortality calculations, 6 had 1 or both diagnosis and 13 one or both CRS-R scores missing (1 and 6 of them died) and were excluded. For the Kaplan–Meier estimator, the seven patients who were missing one or both diagnoses had to be excluded as well. Comparing patients who improved their diagnosis during their hospital stay to patients who did not improve, for the 1-year mortality data the OR of 0.42, 95% CI [0.19–0.92] showed that patients who showed an improvement in diagnosis had a 58% lower probability of dying within the first year after onset. For the 5-year mortality data the OR was 0.33, 95% CI [0.16–0.68], and the probability of dying within 5 years after onset 67% lower for patients who showed an improvement in diagnosis. The median survival was 4,209 days, 95% CI [2361-NA] (139 months, 95% CI [78-NA] or 11.5 years, 95% CI [6.5-NA]) after DoC onset for patients who showed an improvement of DoC category during the hospital stay and 833 days, 95% CI [424–1,586] (27 months, 95% CI [14–52] or 2.3 years, 95% CI [1.2–4.4]) for those without any change in diagnosis. As the survival probability for those who deteriorated did not drop below 50% in this sample, no median survival can be estimated for this group. Figure 8 visualizes this statement.

### 3.7 Epilepsy and status epilepticus

**Figure 9:**
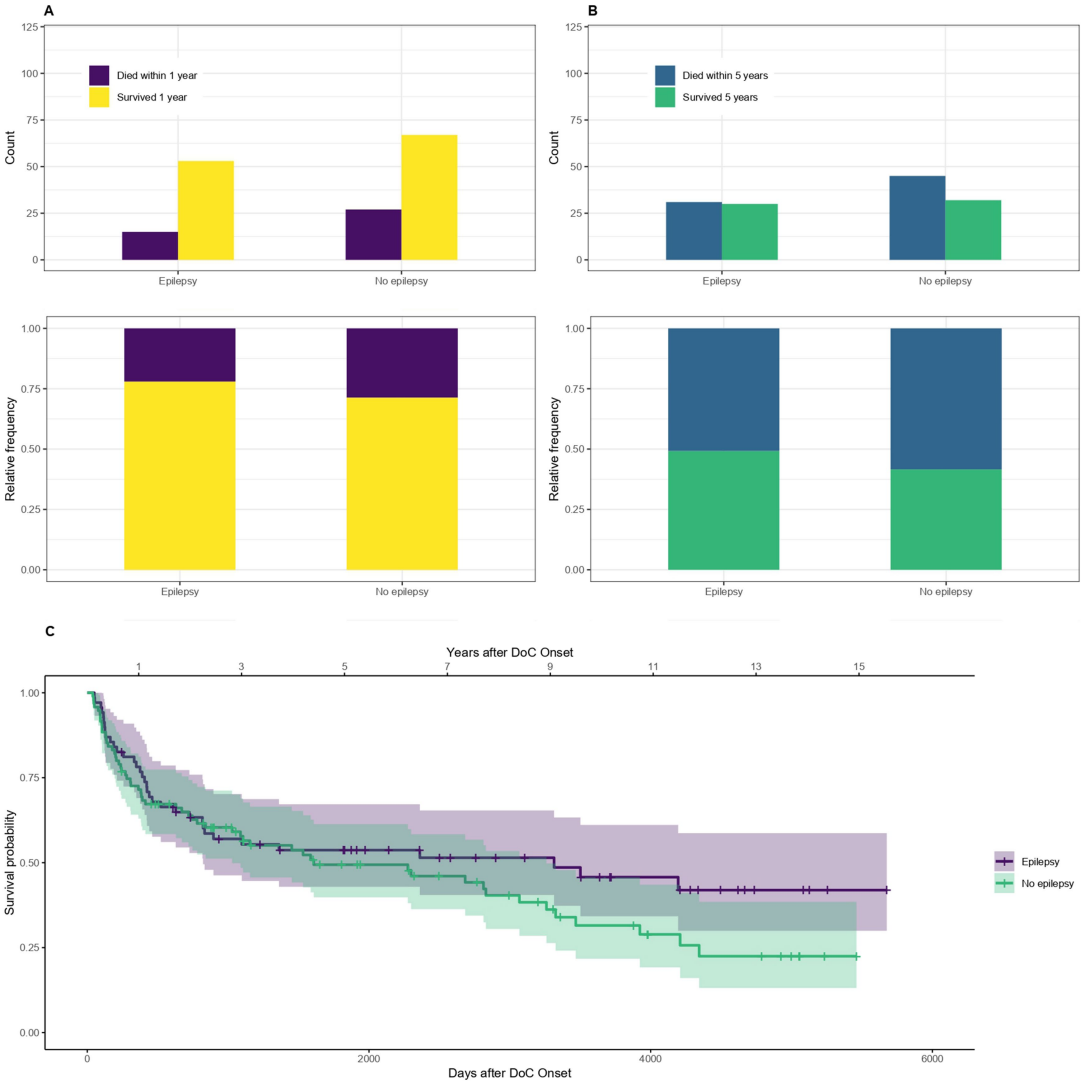
Survival according to epilepsy: absolute and relative counts of patients who suffered from symptomatic epilepsy or a status epilepticus during their hospital stay surviving after 1 **(A)** or 5 **(B)** years after onset of DoC, **(C)** Kaplan–Meier Survival Function according to epilepsy.

The mortality of patients with symptomatic epilepsy or a status epilepticus compared to those without epilepsy is shown in Figure 9. The OR for dying within the first year is 0.7, 95% CI [0.34–1.45] and within 5 years after DoC onset 0.74, 95% CI [0.37–1.45] for patients with compared to without epilepsy. The median survival amounts to 3,314 days, 95% CI [822-NA] (109 months, 95% CI [27-NA] or 9.1 years, 95% CI [2.3-NA]) after DoC onset for patients with epilepsy and 1,608 days, 95% CI [1028–3,260] (53 months, 95% CI [34–107] or 4.4 years, 95% CI [2.8–8.9]) after DoC onset for those without.

### 3.8 Survival time analysis using cox-regression

Table 5 shows the results of the cox-regression. The HR describes how much more likely death occurs if the predictor is present compared to when not (for nominal predictors), or if the value of the predictor is increased by 1 (for metric predictors). A value >1 therefore indicates an increased probability of dying. Summing up, the results of the Cox model point into the same direction as the above-mentioned univariate analyses. Yet, none of the predictors were significant at the adjusted 5 % significance level.

**Table 5:** Results of the cox-regression: evaluation of different predictors on the survival time.

	Hazard ratio (HR)	CI (95%)	1/HR	*p*-value	*p*-value (adjusted*)
Age at onset	1.01	0.99–1.02	1	0.6	1
Sex: men	1.2	0.7–2.05	0.84	0.51	1
Etiology: traumatic	0.96	0.48–1.91	1.04	0.91	1
Etiology: others non-traumatic	1.58	0.84–2.95	0.64	0.16	0.93
Score first CRS-R	0.99	0.95–1.04	1.01	0.79	1
Difference first/last CRS-R	1	0.96–1.05	1	0.9	1

*adjusted according to Bonferroni-Holm.

HR, Hazard Ratio; CI, confidence interval; CRS-R, coma recovery scale revised.

## 4 Discussion

In this community based study, we found a substantially increased mortality in DoC patients compared to the general population. Nevertheless, patients can survive for many years and especially women, younger patients, patients of traumatic etiology and those with a better diagnosis or CRS-R score show a longer median survival.

A comparably high mortality rate in the first year after DoC onset has been reported in various studies (9, 20–22). Whereas a study conducted between 1973 and 1978 reported the death of 75% after 5 years (21), this study showed that only 55.1% of patients die within this timeframe. This is most likely due to improved medical care as not only intensive care and the treatment of complications was enhanced in the recent decades but also the awareness of syndromes like the MCS increased, leading to better care and rehabilitation options for these patients. More recent studies reported that 74% of anoxic DoC patients survived a median time of 16 months (24) and 80.5% of DoC patients survived the first year (23), which is comparable to the 74.1% reported in this study.

Data on incidence of DoC is rare. One study in 2005 estimates the incidence of the vegetative state to be between 0.5 and 2.5/100.000 inhabitants, which would be in line with the incidence of 0.8 UWS patients/100.000 inhabitants found in this study. No recent Austrian numbers are available to compare our findings with and as mentioned in the introduction these numbers can vary significantly between different countries.

In general, comparison with other studies is difficult, not only due to the scarcity of studies on the subject, but also due to insufficient standardization and changes over time. For instance, due to steadily improving emergency and intensive care, more patients survive the initial trauma and reach the state of Doc and also have a better prognosis than they had decades ago.

### 4.1 Age

Besides from the expected influence age has on mortality, further factors could play a role on the finding that younger patients tend to survive longer. Previous studies have shown, that younger age is associated with recovery in DoC patients (31), which, considering that according to our data a better diagnosis is linked to improved survival, could be one factor contributing to the better survival of younger patients in our sample. Comorbidities have also been shown to negatively impact the outcome of DoC patients (32), and as comorbidities tend to be more prevalent in older age, this could also be a contributing factor. A meta-analysis showed that DoC patients of anoxic etiology who died were significantly older than those who survived (24). Moreover, age was found to be an independent predictor of mortality in patients suffering from traumatic brain injury (33).

### 4.2 Sex

The lower proportion of women in our sample could be due to the fact that women are less likely to suffer a traumatic brain injury (34) and have a lower risk for cardiovascular disease (35), which are main causes for DoC. In our study, women survive longer after DoC onset than men. It is well established that in high income countries women tend to live longer, most likely due to biological, environmental and societal factors, even though the gap is getting smaller (36). According to the Statistik Austria, in 2019 the general live expectancy for women in Austria was 84 years and for men 79.3 years (37). However, conflicting findings exist concerning the influence of sex on mortality in DoC patients (22, 38) and a meta-analysis found that sex did not differ between survivors and non-survivors of anoxic DoC (24).

The SMR was more than 21 (1-year mortality) and nine (5-year mortality) times higher for DoC patients than the general population. This is attributable to the severe nature of the syndrome. The higher ratio for the 1-year SMR compared to the 5-year calculations reflects the high mortality within the first year after onset of DoC.

### 4.3 Etiology

This study revealed a better survival for DoC patients of a traumatic etiology compared to those of non-traumatic etiology. Traumatic etiology has been associated with better outcome before (39), whereas non-traumatic etiology was shown to be a risk factor for mortality (22).

### 4.4 First and last diagnosis and CRS-R score

Our data showed that patients with an initial or final diagnosis of UWS have a shorter survival time than patients who are diagnosed as MCS or recovered at the time of first diagnosis or discharge. These findings are in line with previous study results including a meta-analysis (24, 40). Comparing the survival times according to diagnosis, it is evident that the discrepancies between survival of UWS and MCS patients is most notable for the diagnoses at discharge. This is most likely due to the more stable diagnoses at discharge compared to the first diagnosis which are taken earlier, when a change of condition is more likely.

### 4.5 Improvement/deterioration of diagnosis and change of CRS-R score

Patients who improve their diagnosis during the hospital stay have a better survival than those who do not improve or deteriorate. An early improvement can therefore indicate a better long-term survival.

### 4.6 Epilepsy and status epilepticus

The finding that patients who have symptomatic epilepsy or a status epilepticus tend to live longer is very surprising and counterintuitive. However, the CI of the difference between the groups was not statistically significant. Former studies reported worse outcomes for DoC patients with epilepsy (41). It is possible that the retrospective collection of the data on epilepsy lead to some inaccuracy or that the negative impact of epilepsy on the mortality was offset by early recognition and treatment.

### 4.7 Survival time analysis using cox-regression

The Cox-regression revealed no single significant predictor. This might be due to the relatively small sample size and/or substantial between-subject variability.

### 4.8 Limitations

Although the patient sample described in this study is comparably large, it is possible that not all patients in Salzburg County were included and that the incidence is slightly higher than calculated. The catchment area of our hospital includes the Salzburg North area (VR51), in the Salzburg South area (VR52) some patients may have been treated in other hospitals and were therefore not included in the study (selection bias). Nevertheless, the incidence was calculated separately for the Salzburg North area and while we have a selected population for the southern region, the study is population based for the Region VR51 (north). Furthermore, children who suffer from DoC were not included in the study and although these cases are rare, they have been reported (42). Moreover, the study was monocentric and the results cannot be generalized to other parts of the world where different social values, hampered access to emergency and intensive care and various incidences of syndromes leading to DoC (e.g., traumatic brain injury) influence the results. Information bias also has to be considered, however by using the CRS-R as standardized diagnostic tool and hospital records as reliable sources of information, information bias was kept to a minimum.

The collected data is inhomogeneous, especially the time of the first diagnosis and the follow up period until discharge varies considerably between individuals which could distort the findings concerning the impact of the diagnosis on the survival time. However, in clinical practice it is not feasible or would entail a high amount of extra work to align these time points for every patient. Nevertheless, we were able to counteract the problem to a certain degree by applying strict inclusion criteria (DoC diagnosis >28 days and < 6 months after onset). Furthermore, a single CRS-R assessment was used to determine the first and last diagnosis. This could pose a problem in some cases, for instance when a time point was chosen for the assessment where the patient’s vigilance was not the best. Moreover, the diagnosis at the time of death or at the censoring date is unknown, which would help to better understand the long-time prognosis.

Finally, the death statistics provided by the Statistik Austria has limitations too. It is possible that patients moved to another country and changed their nationality, so their deaths would not be registered. Patients whose current address was outside of Austria at DoC onset and who were not of Austrian nationality were therefore excluded from this study. Furthermore, it is possible that the underlying cause of DoC was listed as cause of death in some cases and not the immediate cause of death, which would distort the listing of causes in this study.

## 5 Conclusion

This article gives a rare insight into epidemiological data on DoC patients like incidence and mortality. This will not only improve our understanding of the prognosis of individual DoC patients, but also help estimating and planning for the care expenditure needed for this patient group. Nevertheless more studies in this area are needed, especially from different countries in order to fully understand the epidemiology of DoC. An international registry would dramatically improve the research in this area.

## Data Availability

The datasets presented in this article are not readily available because detailed data on single patients cannot be provided due to data security reasons. Requests to access the datasets should be directed to laura.schnetzer@alumni.pmu.ac.at.
